# Probiotic-based therapeutics for a One Health future: redefining antibiotic dependency to combat antimicrobial resistance

**DOI:** 10.3389/fmicb.2026.1736436

**Published:** 2026-02-12

**Authors:** Veilumuthu Pattapulavar, Sanjai Kumar S, Sathiyabama Ramanujam, Saranyadevi Subburaj, Godwin Christopher John

**Affiliations:** 1 Department of Biomedical Sciences, School of Biosciences and Technology, Vellore Institute of Technology, Vellore, India; 2 Department of Science and Humanities, Karpagam Academy of Higher Education, Coimbatore, India; 3 Department of Biotechnology, Faculty of Engineering (FoE), Karpagam Academy of Higher Education, Coimbatore, India

**Keywords:** AI-driven surveillance, antimicrobial resistance (AMR), ecological therapeutics, microbial resilience, One Health Microbiome Intelligence Framework (OH-MIF), microbiome literacy, probiotics

## Abstract

Antimicrobial resistance (AMR) has become a major One Health concern, affecting the interconnected microbial systems shared by humans, animals, and the environment. Decades of antibiotic-driven control have disturbed ecological stability and contributed to the expansion of the global resistome. This Perspective approaches AMR mitigation through an ecological restoration lens, outlining a three-part strategy that brings together probiotic therapeutics, microbiome-focused public awareness, and integrated surveillance. Probiotics are presented as biologically compatible tools that promote microbial stability through competitive niche occupation, immune support, and environmental biodegradation, thereby reducing selective pressures that favor resistance. In parallel, strengthening microbiome literacy can guide behavioral choices that support stewardship and reduce unnecessary antimicrobial use. The proposed One Health Microbiome Intelligence Framework (OH-MIF) adds a data-driven layer by linking genomic, clinical, agricultural, and environmental information through AI-enabled analytics. Together, these components form an adaptable system that shifts AMR management from reactive dependence on antibiotics toward a more resilient, coexistence-based approach. By aligning ecological interventions with education and policy intelligence, this Perspective positions microbial balance as a practical foundation for sustainable AMR control within broader planetary health goals.

## 1 Introduction

Antimicrobial resistance (AMR) has become one of the One Health Challenges of the 21st century, a crisis that spans human, animal, and environmental systems in a connected ecosystem. The AMR burden is still rising around the world, and it is projected that tens of millions of deaths every year and serious economic damages may occur if the current trends remain the same (Naghavi et al., 2024). This interdependence is characteristic of the One Health framework, where the pathogenic agents and genes move through the clinical, agricultural, and ecological borders with ease, which highlights that no single sector can adequately address AMR alone (Musicha et al., 2024).

Excessive dependence on antibiotics has significantly interfered with the microbial balance in the ecosystems. Broad-spectrum antibiotics can modify gut microbiota in humans and decrease microbial diversity, as well as encourage the colonization of resistant opportunists (John et al., 2024). Long-term antimicrobial growth promoters have been used in livestock, and this has favored resistant strains that are transferred to the food chain (Sachdeva et al., 2024). In the meantime, on an environmental surface (soil and water), antibiotic residues and bacteria that resist them often co-exist, and it is possible to exchange genes through horizontal transfer and mobile genetic elements (Larsson and Flach, 2022; Naccache et al., 2025). According to metagenomic studies, the soil resistome is becoming linked to clinical pathogens, which validates the transfer of genes across habitats as a primary factor in the spread of AMR globally (Zhao et al., 2025). Taken together, these results reveal the mutilation of microbial ecologies by antimicrobial abuse at all scales, from the gut scale to the global biogeochemical cycles. Traditional mitigation strategies have reached a critical impasse. The antibiotic discovery pipeline has stagnated; most new approvals since 2010 belong to existing classes already vulnerable to known resistance mechanisms (Ajose et al., 2024). Stewardship and surveillance programs, while essential, are insufficient to reverse decades of ecological damage (Ifedinezi et al., 2024). The global resistome, once a fragmented collection of local genetic reservoirs, now represents a dynamic network where resistance determinants flow between clinical, agricultural, and natural environments (Fernández-Trapote et al., 2024). The evolutionary and ecological complexity of AMR demands approaches that increasingly support microbial stability rather than relying solely on microbe-directed suppression.

Probiotics are live microorganisms that provide health benefits to the host when administered. They have emerged as biologically compatible approaches for addressing AMR by enhancing microbial homeostasis rather than destroying microbial communities. Clinical and experimental studies reveal that the application of probiotics is able to reduce the impact of antibiotic-associated dysbiosis and decrease the prevalence of antimicrobial resistance genes (John et al., 2024; Wanyama et al., 2025). High-affinity probiotic strains in livestock systems have been reported to control pathogens, including *Salmonella enteritidis* and *Clostridium perfringens*, to improve food safety and reduce the use of antimicrobials (Kosuri et al., 2025; Malli et al., 2025). Synthetic probiotics are also present, and further explore this boundary, as novel structures hold the ability to bind and neutralize the remaining antibiotics of the gut, reducing the selection advantages (Saleh et al., 2025). Nonetheless, research warns that there is a possibility that a certain number of commercial probiotic preparations have transferable resistance genes themselves, which is why they require genomic screenings and regulatory evaluations (Tian et al., 2025).

The One Health framework provides the most consistent perspective through which interventions based on probiotics can be scaled down to sustainability levels. Microbiomes of the human gut, animal intestinal, and environmental systems constitute an extensive system of microbial interchange, and the stability of positive communities in a single area can be extended to others (Fu et al., 2023). Introducing probiotics into One Health systems of governance, which encompass healthcare, veterinary care, food safety, and environmental policy, can serve as a holistic intervention to decrease antibiotic pressure and promote ecological recovery. In addition, epidemiological studies involving metagenomics on a massive scale indicate that both genetic compatibility and environmental connectivity influence the spread of ARGs, and indicate that interventions disrupting these ecological processes could be useful to slow the evolution of AMR (Lund et al., 2025; Napit et al., 2025).

In this context, probiotics represent more than therapeutic supplements; they are ecological stabilizers that can realign microbial interactions across the human-animal-environment interface. The way forward involves a concerted monitoring of the resistome, standardizing the pipelines of probiotic validation, and increasing public awareness to ensure antibiotics remain truly last-resort options. The future against AMR will not be constructed with the stronger antibiotics, but rather with re-engineering microbial symbiosis, with probiotics as ecological allies, based on solid One Health partnership and evidence-based governance.

Ecological restoration, in terms of antimicrobial resistance, does not mean a recession to a pre-antibiotic condition but, instead a quantifiable movement toward microbial stability of human, animal, and ecosystem assemblage. Practically, restoration may be determined by such indicators like recovery of microbial diversity, decreased dominance of resistant taxa, decline in abundance and movement of antimicrobial resistance genes (ARGs), diminished pressure of colonization in pathogens, and diminished dependence on antimicrobial interventions. These metrics have served as a pragmatic interface between the ecological theory and the AMR management to enable the assessment of restoration using microbiome composition biomarkers, resistome dynamics biomarkers, and patterns of antimicrobial use that cross One Health domains.

## 2 Probiotic-based therapeutics: redefining antibiotic dependency across the One Health spectrum

### 2.1 Eradication to equilibrium: probiotics as next-generation antimicrobials

**FIGURE 1:**
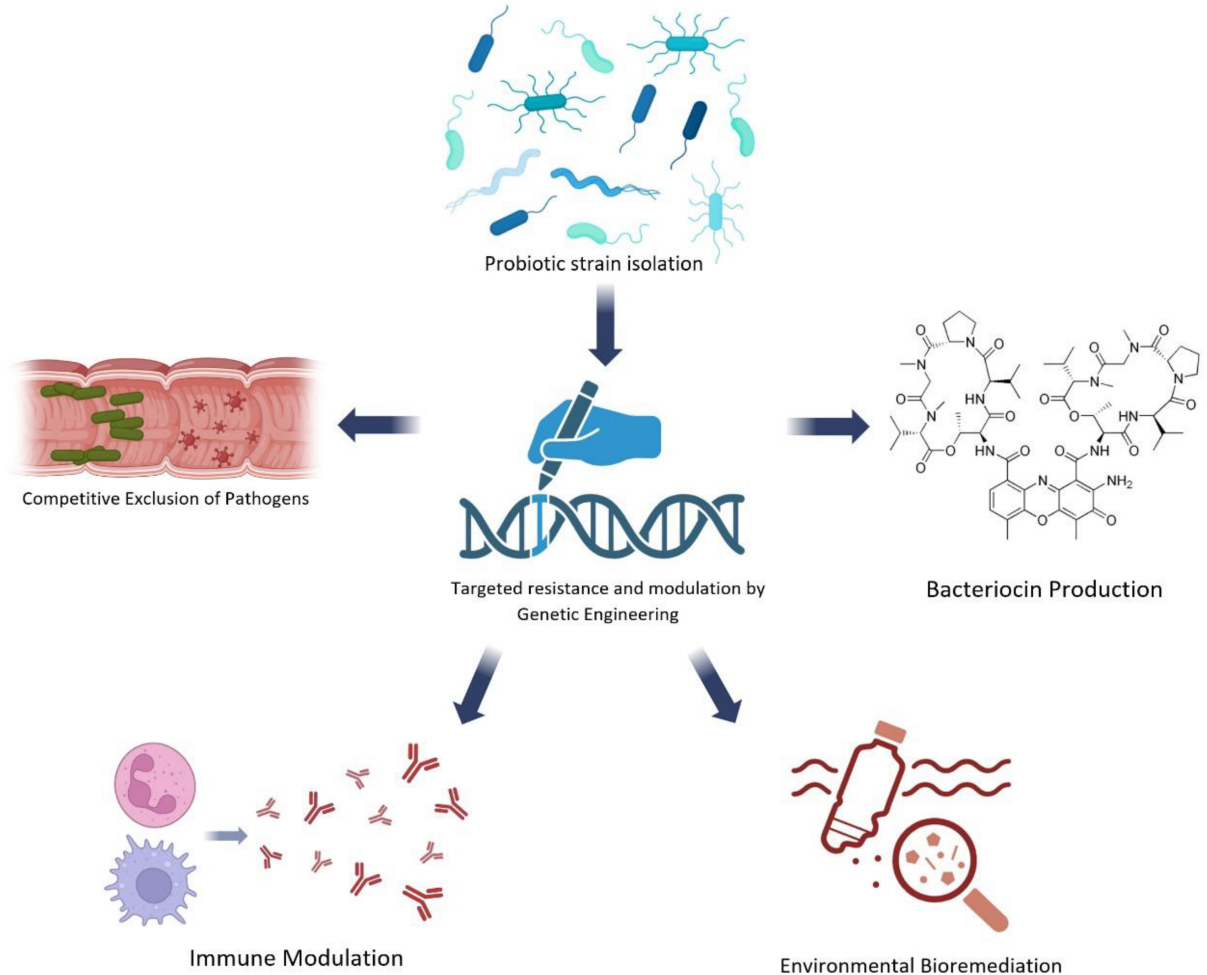
The probiotic pipeline: from isolation to application.

Infection control has historically centered on pathogen elimination, an intervention that views microorganisms as an undesirable threat to be removed, rather than as contributors to ecological balance. Previously considered a life-saving breakthrough, this model of reduction has led to the destabilization of microbial networks and is now contributing to the global increase in antimicrobial resistance (AMR). The emerging paradigm reframes infection control based on ecological therapeutics, which involves the restoration of the microbial balance rather than continuous destruction of them (Ahmed et al., 2024; Blaskovich and Cooper, 2025). In this context, the probiotics represent a next-generation antimicrobial approach designed to coexist rather than compete with microbial ecosystems. Their underlying philosophy is consistent with the One Health ethos, that life security is a matter not of fighting off microbes but of dynamism and equilibrium among the ecosystems. These concepts are further illustrated in the probiotic development pipeline, summarized in Figure 1, which delineates the Probiotic Pipeline: From Isolation to Application.

The process begins with probiotic strain isolation, where beneficial microorganisms are selected from diverse ecological niches such as soil, fermented foods, or the gut microbiome. These isolates are characterized by their safety, adaptability, and functional resilience under host and environmental conditions. Subsequently, genetic engineering enables targeted optimization of these strains. Through synthetic biology and genome editing, specific traits–such as antimicrobial peptide synthesis or resistance modulation–can be fine-tuned to enhance therapeutic efficacy while maintaining biosafety. Such precision tailoring may support the development of next-generation probiotics that align with One Health goals of minimizing resistance transfer. Within the host, probiotics exert protective effects through competitive exclusion of pathogens, occupying ecological niches and preventing opportunistic invaders from adhering to mucosal surfaces or accessing nutrients. They further strengthen this defense by producing bacteriocins–ribosomally synthesized antimicrobial peptides that selectively inhibit pathogens without disturbing commensal microbiota. Beyond pathogen control, probiotics also perform immune modulation, interacting with epithelial and immune cells to reinforce mucosal barrier integrity, stimulate antibody responses, and regulate inflammatory signaling pathways. This dual local and systemic activity promotes overall microbial–host homeostasis. Finally, the pipeline extends beyond clinical use into environmental bioremediation, where probiotic and microbial consortia degrade antibiotic residues and organic pollutants, thereby reducing the environmental resistome. These interlinked mechanisms collectively position probiotics as potential ecological stabilizers–agents that restore microbial equilibrium across human, animal, and environmental systems within the One Health continuum.

Probiotics act through several synergistic pathways in a mechanistic manner, which together may suppress pathogen growth and may inhibit the development of resistance. The competitive exclusion is one of the main strategies according to which probiotics take ecological niches previously occupied by opportunistic pathogens, depriving them of adhesion points and nutrients (Yu et al., 2025). Some of the strains encode bacteriocins, which are ribosomally encoded peptides that affect pathogenic bacteria specifically without affecting the commensal species in general. They also alter gut pH by generating organic acids or intervening quorum-sensing activation of virulence and biofilm formation, in effect silencing virulence resistance-promoting behaviors (Duche et al., 2023). Probiotics also regulate the immune system to maintain the integrity of the mucosal barriers and initiate the secretion of antimicrobial peptides locally, which form an inner system with resistance against infection.

What should, however, be noted is the fact that the majority of the existing probiotics have a transient colonization of the gastrointestinal tract. Their stay is usually temporary in nature, and their functional effects usually fade away on withdrawal. In contrast to resident commensals, a lot of probiotic strains fail to integrate and become long-term ecologically, and their utility is conditional on sustained consumption, affinity to a particular host, and environmental situation. Probiotics, therefore, can no longer be viewed as lasting replenishing agents of disturbed microbiomes, but instead ecological regulators that have the subtle effect of stabilizing microbial communities at times of weakness, like in the face of antibiotic exposure. They mainly provide resistance to selective pressure and buffering of dysbiosis instead of providing a long-term dominance of a set of microbes.

Probiotics have shown promising outcomes in controlled contexts in humans to help alleviate antibiotic-associated dysbiosis, as well as regulate the gut resistome. According to the results of metagenomic studies, probiotics can reduce the prevalence of antimicrobial resistance genes in colonization-permissive individuals, indicating that the impact of probiotics on microbial rebalancing is specific to each individual (Montassier et al., 2021). The future of microbiome biology, newer models of microbiome therapeutics merge microbiome profiling and probiotic selection, moving toward more clinically specific microbial intervention personalization (Abdul Manan, 2025). Based on targeted regulation of metabolic and inflammatory pathways, next-generation probiotics include *Akkermansia muciniphila* and *Faecalibacterium prausnitzii*, which represent a shift in strategy toward the generalized supplementation of probiotics to precision bioengineering (Abouelela and Helmy, 2024; Tiwari et al., 2024).

According to their biological potential, strict anaerobes, including *Akkermansia muciniphila* and *Faecalibacterium prausnitzii*, have considerable translational limitations. They must be grown under hypoxic bioprocessing, encapsulation-specific, and cold-chain logistics are highly controlled, making them cost-prohibitively expensive to produce, in addition to reducing scalability. These limitations have so far limited their use to highly monitored clinical or experimental trials, and they are in practice not practical as an intercessory action at the population level in most areas. Such organisms, in this case, are more about evidence-of-concept precursors of precision microbiome therapeutics aimed at not an immediate use as a population health instrument. Other more viable translational options, such as spore-forming strains, formulated mixtures of defined microbial communities, and postbiotic formulations, may provide more practical translational solutions, especially in low-resource and large-scale agriculture (Abouelela and Helmy, 2024; Tiwari et al., 2024).

Alternatives to antibiotic growth promoters (AGPs) in animal health have been investigated using probiotics, mainly by lightening the burden of pathogens and enhancing the integrity of the gut. Nonetheless, in contrast to antimicrobials, the growth-promoting effects that AGPs have historically played in all of the production systems are not always replicated with probiotics. They are effective depending on the host species, husbandry conditions, diet, and formulation, and trade-offs in economics have continued as a major obstacle to implementation. Based on this, probiotics cannot be considered as direct functional substitutes of AGPs; rather, they are risk-reducing and resilience-promoting solutions, which facilitate a slow transformation in the habit of using antimicrobials in the context of stewardship. The experimental models have also shown that probiotic supplementation has been associated with decreased pathogen load and enhanced the morphology of the gut, and little selection pressure is induced by the use of antimicrobials. Nevertheless, the commercial preparations contain some remnants of transferable resistance genes, such as tetS and APH (3)-Ia, which highlights the significance of rigorous genomic safety assays (Kerek et al., 2024). Claiming that probiotic-based sanitation systems have proven to be ecologically promising, long-term hospital trials using *Bacillus subtilis* and *B. velezensis* consortia have demonstrated genomic stability under studied conditions, with neither virulence nor resistance determinants, making them environmentally safe and capable of minimizing pathogen bioburden (Bini et al., 2025). These results corroborate the point that probiotic interventions can act safely within One Health realms when they are duly validated.

Beyond host-associated benefits, probiotics also contribute to environmental stability, particularly through mechanisms that support bioremediation. Such systems can also cause the environmental resistome to become disconnected by surpassing resistant bacteria and restricting the spread of antibiotic resistance genes (ARGs) horizontally (Klümper et al., 2025). Incorporation of such microbial solutions into wastewater treatment and runoff control of agricultural enterprises would help to significantly reduce the spread of resistance on the ecosystem level.

Innovation leads to frontiers that create probiotics in programmable therapeutics and not just in natural systems. Synthetic biology is being used to create engineered probiotics, which can be designed to release particular molecules or combat pathogens locally (Zhang et al., 2025). CRISPR-based reagents provide a chance to specifically remove resistance plasmids, providing a nice biotechnological breadth of path to reverse resistance dissemination (Liu et al., 2023; Rahmati et al., 2025). Similar antimicrobial activities are supported by parallel improvements in non-viable microbial components for enhancing microbial health, known as postbiotics, which do not provoke regulatory or stability complications associated with live cultures (Amobonye et al., 2025; Calvanese et al., 2025). Together, these advancements indicate the coming together of the science of microbiomes, genomics, and biotechnology into one therapeutic frontier.

The next stage of infection management may involve not only the development of the biochemical arms race but also reprogramming ecological resilience. Precursors and derivatives of probiotics relate to such transformation: adaptive, sustainable, and integrative agents, and they work along the animal-environment-human continuum. Representative probiotic strains validated for AMR mitigation across human, animal, and environmental contexts are summarized in Table 1. Probiotic-based therapeutics can reimagine the role of combating AMR not by dominance, but by balance, switching to a microbial coexistence model. Such ecosystem-level validation demands harmonized policy frameworks that connect laboratory efficacy with field sustainability–central to the One Health vision of balanced microbial governance.

**TABLE 1:** Experimentally validated probiotic strains and their ecological mechanisms in mitigating antimicrobial resistance within the One Health spectrum.

Probiotic strain	Mechanism of action	Outcome	One Health relevance	References
*Akkermansia muciniphila*	Modulates host mucosal immunity and regulates inflammatory pathways to maintain microbial balance	Enhances gut barrier integrity and reduces dysbiosis associated with antibiotic use	Represents a next-generation probiotic model for precision microbiome therapeutics	Tiwari et al., 2024
*Faecalibacterium prausnitzii*	Produces anti-inflammatory metabolites and reinforces epithelial tight junctions	Restores microbial homeostasis disrupted by antibiotic exposure	Supports gut ecosystem stability and prevents colonization by resistant opportunists	Abouelela and Helmy, 2024
*Bacillus subtilis*	Produces extracellular enzymes and biosurfactants with antimicrobial activity; forms stable spores for environmental persistence	Reduces pathogen bioburden on hospital and animal surfaces	Demonstrated safe and stable genomic profile with no virulence or resistance determinants in probiotic sanitation trials	Bini et al., 2025
*Bacillus velezensis*	Exhibits competitive exclusion and degradation of antibiotic residues in environmental systems	Minimizes ARG (antibiotic resistance gene) exchange and environmental contamination	Enhances ecological bioremediation and supports microbiome resilience in One Health ecosystems	Klümper et al., 2025

The strongest evidence of probiotic interventions in AMR reduction is best seen in situations where antibiotics interfere with the homeostasis of microbes and not in the reversal of those at the population level. The meta-analyses and controlled studies all indicate that probiotics prevent antibiotic-associated dysbiosis, prevent the overgrowth of pathogens and alter the resistome composition in a host- and antibiotic-specific way. Notably, it is the role of decreased selective pressure, competition to occupy a niche, and stabilization of commensal communities that mediate these effects, as opposed to direct eradication of resistance determinants. In that regard, probiotics have to be discussed as pressure-reducing and resilience-building mechanisms which supplement, yet do not substitute antimicrobial interventions in the framework of integrated stewardship approaches.

### 2.2 Awareness as activation: advancing microbiome literacy against AMR

Beyond biology, AMR persistence reveals a cultural dimension, where misinformation, policy gaps, and behavioral inertia intersect. Antimicrobial resistance (AMR) has often been discussed as a pharmacological crisis caused by microbial evolution and clinical abuse, but its persistence also reflects underlying cultural and behavioral challenges. Nevertheless, despite all the campaigns, the global societies perceive the use of antibiotics in a biased manner, where instead of looking at the systems, microbiomes, and ecosystems, they look at medications and pathogens. Such narrowing down keeps reactionary policies and impedes the change of behavior. Research shows that the incorrect beliefs are still present even in developed countries, where most people still hold the belief that resistance develops within the human body or that antibiotics help them overcome viral diseases (Molan et al., 2025). These misconceptions continue to perpetuate self-prescription, lack of compliance, and abuse across all markets. An overview of the current limitations in antimicrobial resistance management, along with emerging solutions based on probiotics, microbiome literacy, and One Health–aligned surveillance, is presented in Table 2.

**TABLE 2:** Addressing current limitations in antimicrobial resistance management through probiotics, microbiome literacy, and One Health–aligned surveillance.

Current limitation in AMR management	Probiotic-based contribution	Microbiome literacy contribution	OH-MIF/surveillance contribution
Heavy reliance on antibiotics for infection control	Reduces selective pressure through competitive exclusion and microbiome stabilization	Encourages acceptance of non-antibiotic interventions	Monitors shifts in resistance linked to intervention strategies
Antibiotic-associated dysbiosis and pathogen overgrowth	Restores commensal balance and suppresses opportunistic pathogens	Improves adherence to appropriate antibiotic use	Tracks microbiome and resistome recovery trends
Fragmented surveillance across sectors	Supports ecological resilience across host and environment	Promotes cross-sectoral stewardship awareness	Integrates clinical, agricultural, and environmental data
Limited monitoring of ARG mobility	Reduces environmental and host-level resistance amplification	Increases awareness of environmental AMR pathways	Enables early detection of ARG dissemination patterns
Low public engagement in AMR mitigation	Provides tangible alternatives to antibiotics	Translates microbiome science into behavioral change	Aligns data-driven insights with policy responses

To make microbiome literacy operational, principles of microbial ecology should be incorporated into existing public health and governance structures rather than creating parallel educational systems. Effective implementation channels involve antimicrobial stewardship programs, school and medical training, in veterinary extension programs aiming to reduce growth promoters in antimicrobials, and citizen-science engagement practices that involve communities in microbiome awareness. These methods have been linked to a higher rate of antibiotic adherence, a decreased level of self-medication, and an enhanced acceptance of non-antibiotic measures. Microbiome literacy, by providing abstract microbiome notions into behavioral norms, works effectively as a pragmatic upstream intervention by strengthening antimicrobial stewardship along One Health sectors.

However, recent articles have termed AMR a Silent Pandemic, where ignorance, as opposed to biology itself, promotes its transmission (Ahmed et al., 2024). These gaps are not limited to the general population; policymakers, clinicians, and farmers also demonstrate significant awareness deficits present in policymakers, clinicians, and farmers, and they fragment accountability along the One Health axis (Ahmed, 2025). This cycle needs to be addressed by turning awareness into microbiome literacy, or a mutual perception of dependence and ecological strength on microbes (Altevogt et al., 2025).

This shift is testified by educational innovations. The Microbiome and Health web-based course also helped enhance a deeper understanding of microbial ecology and sustainability in different learners (Schweitzer et al., 2025). Microbial literacy can be normalized through embedding this kind of content into school curricula, veterinary education, and health education to produce individuals who understand microbial coexistence rather than viewing microbes as adversaries.

The programs of citizen science have even more democratized the study of the microbiome. Citizen science platforms now allow communities to study their gut microbiomes, leading to transparency and inclusion, which are consistent with cross-sectoral frameworks of surveillance (Lappan et al., 2024; Vallejo-Espín et al., 2025). Social media further serves as an amplifier: campaigns and storytelling based on inclusive messages, like the one claiming that microbiological science is translated into accessible public messages, which is the message that reads like protect your microbes, protect your future.

Yet, literacy should be involved in systemic reform. One Health stewardship model stresses that education alone is insufficient without policy frameworks that reinforce responsible antimicrobial usage (Altevogt et al., 2025). To link microbial ecology to sustainability, environmental education must focus on ARG mobility by means of soil and water systems (Klümper et al., 2025).

Microbiome literacy is a behavioral intervention when supported through education, governance, and culture. Well-informed communities are more compliant with stewardship interventions, less prone to misuse of antibiotics, and more tolerant of alternative forms of treatment like probiotics. Agricultural systems also have awareness that is connected to less use of antimicrobial growth promoters and more that will open to probiotics feed intervention (Kerek et al., 2024). This cultural shift is both behavioral and ecological, moving human activity toward coexistence rather than conflict with microbes.

### 2.3 One Health Microbiome Intelligence Framework (OH-MIF): unifying surveillance and data integration

One Health Microbiome Intelligence Framework (OH-MIF) is suggested as a conceptual surveillance framework intertwining heterogeneous data pertinent to AMR dynamics, as opposed to a technological platform that can be deployed. The core data inputs are clinical information (antimicrobial prescriptions, resistance profile, patient microbiome profile), genomic and metagenomic information (ARG abundance, mobility marker, resistome composition), agricultural information (antimicrobial and probiotic use in livestock systems), and environmental information (wastewater, soil, and surface water resistome). In this context, AI-driven analytics can be used to detect patterns, stratify ecological risk, and create advanced warning signals of the development of resistance instead of discussing clinical or regulatory choices. The most common ones are standardizing data used in sectors, the capability to enable any one of the surveillance systems to work together, anonymity to balance privacy, and compliance with governance among the national and international agencies.

Global AMR surveillance remains fragmented, with the clinical data mostly prevailing, with less integration of the agricultural, environmental, or probiotic knowledge. Existing systems like the GLASS (Global Antimicrobial Resistance and Use Surveillance System) of WHO or other sets of sector surveillance provided by FAO (Food and Agriculture Organization) and OIE (now termed as World Organization for Animal Health) rarely incorporate probiotic performance or microbiome homeostasis indicators (Lappan et al., 2024; Vallejo-Espín et al., 2025). To address this gap, a One Health Microbiome Intelligence Framework (OH-MIF) is proposed, which is a unified system integrating genomic, clinical, agricultural, and environmental microbiome datasets for AMR management. However, implementing such a system requires addressing challenges such as data standardization across countries, interoperability of heterogeneous databases, data privacy protections, and the computational resources needed for large-scale microbiome analytics.

This model would operate with the help of cloud-based databases that can track the safety of probiotic strains, ARG (Antibiotic Resistance Gene) transmission, and ecological performance (Kerek et al., 2024; Zavišić et al., 2023). AI-driven correlation mapping would be able to connect the usage patterns of probiotics and the local trends of AMR (Abdul Manan, 2025), whereas integrated metagenomic pipelines would help track the flow of ARG through the hospital, farm, and wastewater systems (Altevogt et al., 2025; Klümper et al., 2025).

Policy reform is necessary to supplement data integration. A Global Request for Competitive Standing (RCS) system for probiotics, such as Genomic validation, AMR risk profiling, and biosafety certification, would provide the ecological stability and avoid the spread of resistance unintentionally (Kerek et al., 2024; Zavišić et al., 2023). These standards would be institutionalized through intersectoral cooperation of ministries, agricultural boards, and environmental agencies in the process of national AMR action plans.

The OH-MIF is designed to integrate currently isolated biotherapeutic data streams into a coherent AMR mitigation infrastructure. It may enable monitoring microbial dynamics across systems in real-time through standardized data streams, continuous feedback control, and AI-enhanced analytics. This model would harmonize surveillance as well as support adaptive policymaking. The model would strengthen the connection between microbial ecology and the ultimate outcomes of public health.

**FIGURE 2:**
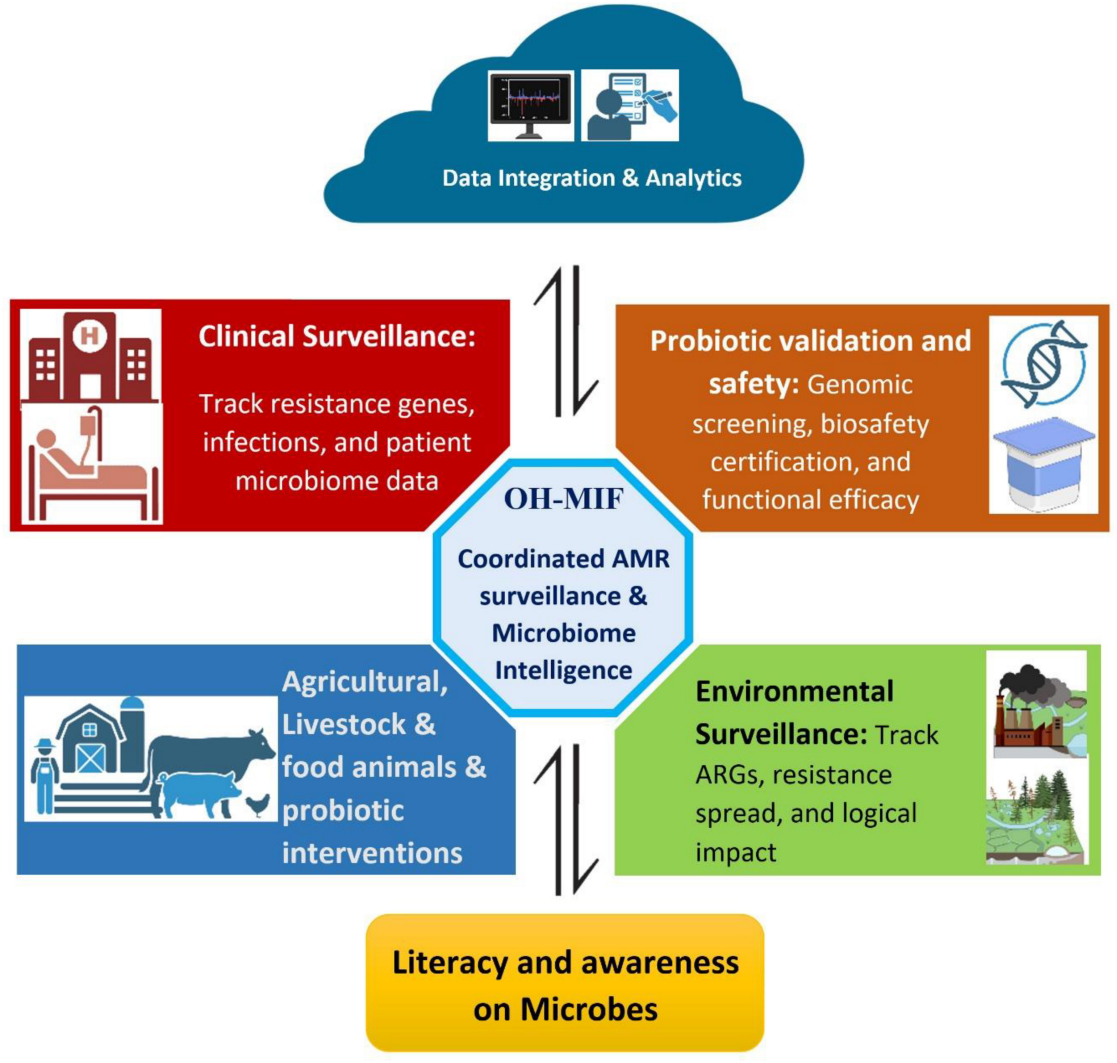
The One Health Microbiome Intelligence Framework (OH-MIF) illustrates an integrated system for coordinated AMR surveillance and microbiome intelligence. At its center, the OH-MIF hub interacts with the Data Integration and Analytics cloud, which combines metagenomic and genomic datasets for AI-driven predictive AMR mapping across hospitals, farms, and wastewater systems, as well as promoting literacy and awareness about microbes among the public. Surrounding this hub are four key components: the Clinical Surveillance module tracks resistance genes, infections, and patient microbiome data to inform adaptive health policies; the Agricultural and Livestock sector monitors antimicrobial use and probiotic interventions to reduce resistance emergence; the Environmental Surveillance component tracks ARG dissemination and the ecological impact of antimicrobial residues; and the Probiotic Validation and Safety unit ensures genomic screening, biosafety certification, and functional efficacy of microbial formulations. Collectively, these components form a unified intelligence network that enables real-time monitoring, prediction, and control of AMR within a One Health context.

Eventually, the future requires probiotics and AMR screening not to develop side by side but synchronously. Coordinated microbiome intelligence can help transform fragmented surveillance data into coherent, actionable insights that support microbial coexistence. Institutionalized information-sharing and environmental integration may enable a shift from reactive response toward anticipatory AMR management and strengthened microbial resilience. The operational structure of this model is depicted in Figure 2, highlighting the integration of clinical, agricultural, and environmental datasets into a unified intelligence framework.

The OH-MIF is set to support the current AMR monitoring programs, not to dissolve them, e.g., the WHO Global Antimicrobial Resistance and Use Surveillance System (GLASS) and FAO-WHO-WOAH Tripartite frameworks. Although the existing systems are mainly aimed at monitoring the prevalence of resistance and antimicrobial usage, OH-MIF builds on the same platform by including the measures of microbiome stability, probiotic safety endorsement, and ARG motility between environmental reservoirs. Placing OH-MIF as part of an established governance framework can increase policy relevance and help become part of the national AMR action plans without further redundancies in surveillance.

## 3 Discussion and future directions

Although probiotic-based interventions have strong ecological benefits, they cannot be considered the answer to antimicrobial resistance. Their impacts can be highly situational, temporal, and inadequate on their own to counteract the dynamics of deep-seated resistance on a rigor mortis-level. Their global implementation is inhibited by structural barriers like the complexity of manufacturing, regulatory heterogeneity, cost, and erratic field performance. Hence, should be laid out as auxiliary products in combined steward plans along with antibiotics, vigilance, and policy changes without substituting the latter. The recognition of them as ecological pressure-reduction means as opposed to curative substitutes puts their realistic abilities into harmony with their real system-level worth.

A combination of probiotic-based therapeutics, the concept of awareness with the implementation, and the coordinated surveillance can be referred to as the triad of successful antimicrobial resistance (AMR) management in One Health. Probiotics hinder the growth of the pathogens as well as reducing resistance by assuming competitive exclusion, production of bacteriocins, immune modulation, and environmental bioremediation (Duche et al., 2023; Klümper et al., 2025; Yu et al., 2025). In line with this, microbiome literacy turns communities into active participants in microbial stewardship rather than passive recipients of information, which encourages behaviors that support ecological well-being (Ahmed et al., 2024; Altevogt et al., 2025; Schweitzer et al., 2025). The joint monitoring, by the One Health Microbiome Intelligence Framework (OH-MIF), involves the streams of clinical, environmental, and probiotic data, which are then coordinated to monitor resistance genes and offer adaptive responses (Kerek et al., 2024; Lappan et al., 2024).

Policy translation also needs interdisciplinary financing and regulatory programmes, guaranteeing the safety of probiotics and other necessary genetic screening processes, and control of transferable resistance genes (Kerek et al., 2024; Zavišić et al., 2023). The safety can be preserved by introducing programmable probiotics and postbiotics, with the primary aim of increasing the scope of interventions.

Further studies are required to validate the effects of probiotics in real-world scenarios. To determine their impact on resistance dynamics, microbiome stability, and functional health outcomes in such precision microbiome therapeutics, long-term ecological trials in hospitals, farms, and natural ecosystems are necessary (Bini et al., 2025; Montassier et al., 2021). Priority areas include multi-year hospital-based microbiome monitoring, livestock system trials that track ARG flow from feed to manure, and watershed-level environmental studies that assess probiotic impacts on soil and water resistomes.

Wider community adoption remains crucial. Major barriers include misinformation, low microbial literacy, cost concerns, and limited regulatory clarity around next-generation probiotics. Targeted public education, integration into national stewardship campaigns, and transparent safety frameworks can improve public trust and adoption. Incorporating probiotics into health programs, promoting citizen-science participation, and strengthening public education can normalize microbial stewardship and decrease dependency on antibiotics, and promote eco-resilience (Schweitzer et al., 2025; Vallejo-Espín et al., 2025).

In conclusion, the traditional microbial conflict thinking must give way to frameworks grounded in coexistence. A way out of resistance to resilience can be Probiotic-based therapeutics, literacy, and integrated surveillance. The One Health approach brings together research, policy, and community strategy that positions microbial coexistence as a central operational principle in global health.

## 4 Conclusion

Antimicrobial resistance (AMR) represents not only a clinical challenge but also an ecological disruption. Restoring this equilibrium will likely require a unified One Health strategy that integrates probiotic-based therapeutics, microbiome literacy, and intelligent surveillance into a single, adaptive system. Probiotics offer sustainable biological solutions that realign microbial networks across humans, animals, and environments. When supported by microbiome-aware education and AI-driven data integration, these interventions shift AMR management from reactive containment toward proactive resilience. The proposed One Health Microbiome Intelligence Framework (OH-MIF) and literacy-driven stewardship exemplify a shift from reliance on antimicrobials toward more ecology-aligned governance. By merging microbial science, social awareness, and policy intelligence, the global health community offers a pathway to redefining the fight against resistance–from attempting to eliminate microbial life toward managing it through informed coexistence.

## Data Availability

The raw data supporting the conclusions of this article will be made available by the authors, without undue reservation.
